# Development of Targeted siRNA Nanocomplexes to Prevent Fibrosis in Experimental Glaucoma Filtration Surgery

**DOI:** 10.1016/j.ymthe.2018.09.004

**Published:** 2018-09-11

**Authors:** Owen Fernando, Aristides D. Tagalakis, Sahar Awwad, Steve Brocchini, Peng T. Khaw, Stephen L. Hart, Cynthia Yu-Wai-Man

**Affiliations:** 1 National Institute for Health Research (NIHR) Biomedical Research Centre at Moorfields Eye Hospital NHS Foundation Trust and UCL Institute of Ophthalmology, London EC1V 2PD, UK; 2 King’s College London, London SE1 7EH, UK; 3 Experimental and Personalised Medicine Section, Genetics and Genomic Medicine Programme, UCL Great Ormond Street Institute of Child Health, London WC1N 1EH, UK; 4 UCL School of Pharmacy, London WC1N 1AX, UK; 5 Department of Biology, Edge Hill University, Ormskirk L39 4QP, UK

**Keywords:** nanoparticle, siRNA, targeting peptide, glaucoma, fibrosis

## Abstract

RNAi induced by double-stranded small interfering RNA (siRNA) molecules has attracted great attention as a naturally occurring approach to silence gene expression with high specificity. The myocardin-related transcription factor/serum response factor (MRTF/SRF) pathway is a master regulator of cytoskeletal gene expression and, thus, represents a promising target to prevent fibrosis. A major hurdle to implementing siRNA therapies is the method of delivery, and we have, thus, optimized lipid-peptide-siRNA (LPR) nanoparticles containing MRTF-B siRNAs as a targeted approach to prevent conjunctival fibrosis. We tested 15 LPR nanoparticle formulations with different lipid compositions, surface charges, and targeting or non-targeting peptides in human conjunctival fibroblasts. *In vitro*, the LPR formulation of the DOTMA/DOPE lipid with the targeting peptide Y (LYR) was the most efficient in *MRTF-B* gene silencing and non-cytotoxic compared to the non-targeting formulation. *In vivo*, subconjunctival administration of LYR nanoparticles containing MRTF-B siRNAs doubled bleb survival in a pre-clinical rabbit model of glaucoma filtration surgery. Furthermore, MRTF-B LYR nanoparticles reduced the *MRTF-B* mRNA by 29.6% in rabbit conjunctival tissues, which led to significantly decreased conjunctival scarring with no adverse side effects. LYR-mediated delivery of siRNA shows promising results to increase bleb survival and to prevent conjunctival fibrosis after glaucoma filtration surgery.

## Graphical Abstract

**Figure:**
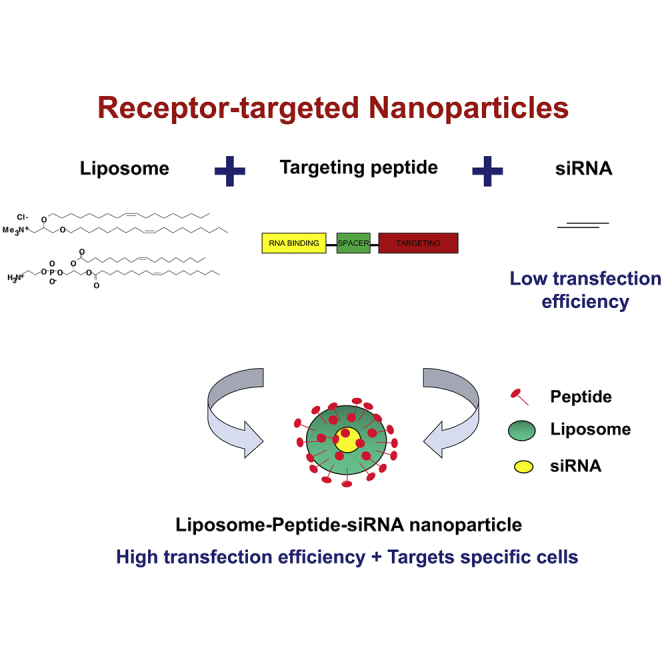

## Teaser

RNAi induced by double-stranded, small interfering RNA (siRNA) is a naturally occurring approach to silence gene expression with high specificity. Yu-Wai-Man and colleagues show that lipid-peptide-siRNA (LPR) nanoparticles significantly increase bleb survival and prevent conjunctival fibrosis in a pre-clinical model of glaucoma filtration surgery.

## Introduction

Glaucoma is the leading cause of irreversible blindness in the world, currently affecting over 60 million people worldwide, and is estimated to rise to 76 million by 2020 and to nearly 112 million by 2040.^1^ Postoperative subconjunctival and episcleral fibroses represent the critical determinant of the long-term surgical outcome and intraocular pressure after glaucoma filtration surgery (GFS).^2^ Mitomycin-C (MMC) is an antimetabolite drug that is used to modulate wound healing in glaucoma surgery and acts by inhibiting DNA synthesis and causing widespread apoptosis.^3^ Serious vision-threatening side effects have been associated with the use of MMC; namely, a 5% risk of hypotonous maculopathy,^4^ severe infection,^5^ corneal melting and perforation,^6^ and scleral calcification.^7^ In addition, some patients still scar and fail surgery, despite antimetabolite therapy. There is, thus, a large unmet need for alternative agents with more targeted physiological effects and less cytotoxicity.

Serum response factor (SRF) is a ubiquitous transcription factor and a master regulator of cytoskeletal gene expression,^8^, ^9^ including many genes involved in fibrosis.^10^, ^11^, ^12^ The Myocardin-Related Transcription Factor (MRTF) family is one of the principal families of signal-regulated SRF co-activators.^9^, ^13^ The two MRTF family members, MRTF-A and MRTF-B, are regulated by cytoskeletal dynamics and respond to variations in the cellular concentration of G-actin, to which they bind through N-terminal RPEL motifs.^14^, ^15^ The MRTF/SRF pathway plays a key role in myofibroblast activation and has been linked to ocular,^16^, ^17^, ^18^, ^19^ vascular,^10^ skin,^11^ and lung fibrosis.^12^

Small interfering RNAs (siRNAs) are double-stranded RNA molecules 20–25 nt long that regulate gene expression by degrading mRNA targets specifically, thereby leading to gene silencing.^20^ We have previously shown that cationic lipid (L)-peptide (P)-siRNA (R) (LPR) nanocomplexes represent an efficient delivery system for *MRTF-B* gene silencing in human conjunctival fibroblasts.^18^ The LPR formulation used *in vitro* may not be suitable for *in vivo* application, as cationic nanocomplexes can display poor tissue penetration, cause non-specific binding to cells, interact with serum proteins, and lead to inflammation. Unlike cationic nanocomplexes, anionic nanocomplexes are resistant to aggregation in the presence of serum^21^ and can achieve significant gene silencing in delivering siRNA.^22^ Cholesterol is a non-charged helper lipid that plays a role in many cellular membrane-related events, such as membrane fusion and endocytosis, and introducing cholesterol as a component of certain DNA-RNA carriers can increase their hydrophobic stability and improve transfection *in vivo* compared to carriers not containing cholesterol.^23^, ^24^ PEGylation can also reduce aggregation due to binding of serum proteins and can increase the receptor-targeted specificity. In the case of anionic nanocomplexes, PEGylation can further enhance the transfection efficiency in cells.^21^ Thus, we optimized LPR formulations by varying the lipid composition to confer different surface properties, including surface charge and PEGylation to enhance their *in vivo* biocompatibility. We also evaluated the peptide targeting specificity in LPR nanocomplexes in transfections of human conjunctival fibroblasts. Finally, we investigated the safety and efficacy of MRTF-B siRNA delivered by targeted LPR nanoparticles on bleb survival and conjunctival scarring in a pre-clinical rabbit model of GFS.

## Results

### Development and Biophysical Characterization of Cationic and Anionic Nanoparticles

**Figure 1:**
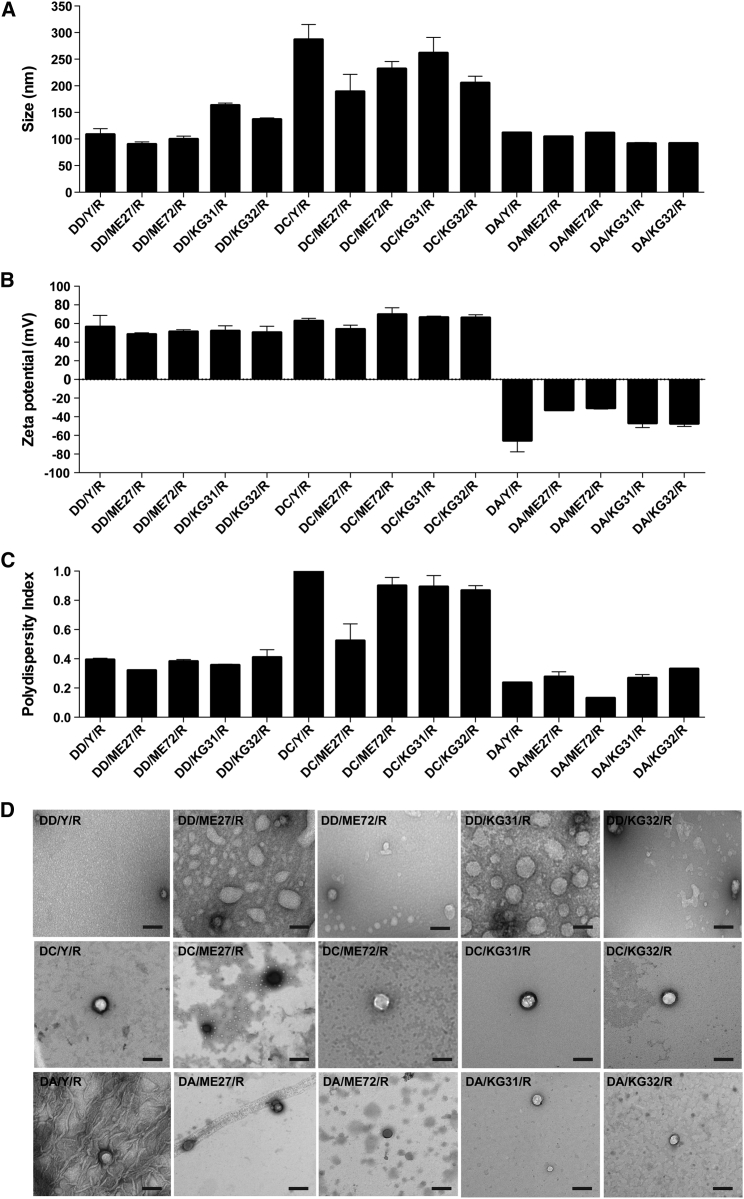
Biophysical Characterization of Multiple LPR Nanoparticle Formulations (A) Size in nanometers. (B) Zeta potential in millivolts. (C) Polydispersity index. Results represent mean ± SEM. (D) Negative-staining transmission electron microscopy. Scale bars, 200 nm. DD, DOTMA/DOPE; DC, DOTMA/DOPE + cholesterol; DA, anionic DOPG/DOPE + PEG; targeting peptides are Y, ME27, KG31, and KG32; non-targeting peptide is ME72; R, siRNA.

We developed and characterized 15 LPR formulations using three types of liposomes (DD [DOTMA/DOPE], DC [DOTMA/DOPE + cholesterol], and DA [anionic DOPG/DOPE + polyethylene glycol; PEG]), five types of peptides (targeting [Y, ME27, KG31, and KG32]) and non-targeting [ME72]), and siRNA (R). LPRs containing DD had a mean size (±SEM) of 119.5 ± 7.3 nm (Figure 1A). Targeting DD/Y/R (108.3 ± 6.4 nm) and DD/ME27/R (89.9 ± 2.7 nm) nanoparticles were similar in size to the non-targeting DD/ME72/R (99.7 ± 3.4 nm). Targeting DD/KG31/R (163.1 ± 2.5 nm) and DD/KG32/R (136.6 ± 1.7 nm) nanoparticles were slightly larger in size. LPRs containing DA had a mean size of 102.1 ± 2.4 nm and were similar in size to DD-containing LPRs. However, LPRs containing DC were significantly larger in size (234.7 ± 11.0 nm) than LPRs containing DD or DA.

LPRs containing DD and DC were strongly cationic and had a mean charge (±SEM) of 51.5 ± 1.7 mV and 63.7 ± 1.8 mV, respectively, whereas LPRs containing DA were anionic (−44.4 ± 3.6 mV) (Figure 1B). LPRs containing DD and DA nanoparticles also had relatively low polydispersity indices (PDIs) of 0.37 ± 0.01 and 0.25 ± 0.02, respectively (Figure 1C). However, DC-containing LPRs had a high PDI of 0.84 ± 0.05. PDIs greater than 0.3 are indicative of multiple populations rather than the ideal monodisperse population. All LPR nanoparticle formulations were spherical in morphology using negative-staining transmission electron microscopy (TEM) (Figure 1D). The bigger size of DC-containing LPRs compared to those containing DD or DA was also verified by TEM.

### Enhanced Silencing Effect in Human Conjunctival Fibroblasts Transfected with Receptor-Targeted Nanoparticles

**Figure 2:**
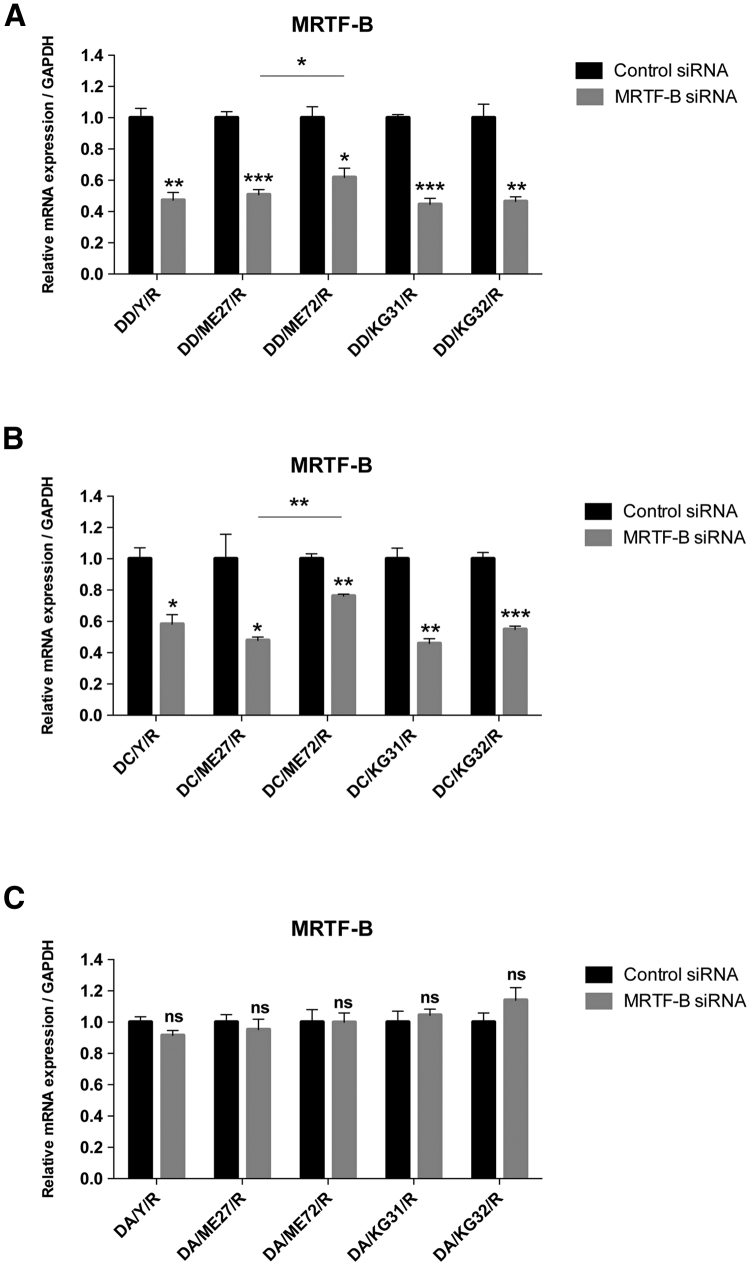
*In Vitro* Transfections of Human Conjunctival Fibroblasts and *MRTF-B* Gene Silencing by LPR Nanoparticles (A) LPRs containing DOTMA/DOPE (DD). (B) LPRs containing DOTMA/DOPE + cholesterol (DC). (C) LPRs containing anionic DOPG/DOPE + PEG (DA). mRNA levels were normalized relative to that of GAPDH, and the results represent mean ± SEM for triplicate experiments. *p < 0.05; **p < 0.01; ***p < 0.001. ns, not significant.

For LPRs containing DD, the silencing efficiency on the *MRTF-B* gene in human conjunctival fibroblasts was significantly increased with the use of targeting peptides (Y: 52.7%, p = 0.002; ME27: 49.1%, p = 0.0006; KG31: 55.4%, p = 0.0002; KG32: 53.5%, p = 0.004) compared to the non-targeting peptide (ME72: 38.1%, p = 0.014) (Figure 2A). Similarly, for LPRs containing DC, the silencing efficiency of the *MRTF-B* gene was also significantly higher with the use of targeting peptides (Y: 41.8%, p = 0.011; ME27: 52.1%, p = 0.022; KG31: 54.1%, p = 0.002; KG32: 45.1%, p = 0.0006) compared to the non-targeting peptide (ME72: 23.7%, p = 0.002) (Figure 2B). There were statistically significant differences in *MRTF-B* gene expression between the ME27 and ME72 peptides containing LPR formulations with DD (p = 0.020) (Figure 2A) and with DC (p = 0.002) (Figure 2B). ME72 contains a scrambled sequence of the ME27 targeting ligand, and this result thus supports the targeting effect of the ME27 peptide. However, targeting and non-targeting DA-containing LPRs did not have any statistically significant effect on *MRTF-B* gene silencing in human conjunctival fibroblasts (Figure 2C).

### Receptor-Targeted LYR Nanoparticles Display Low Cytotoxicity in Human Conjunctival Fibroblasts

**Figure 3:**
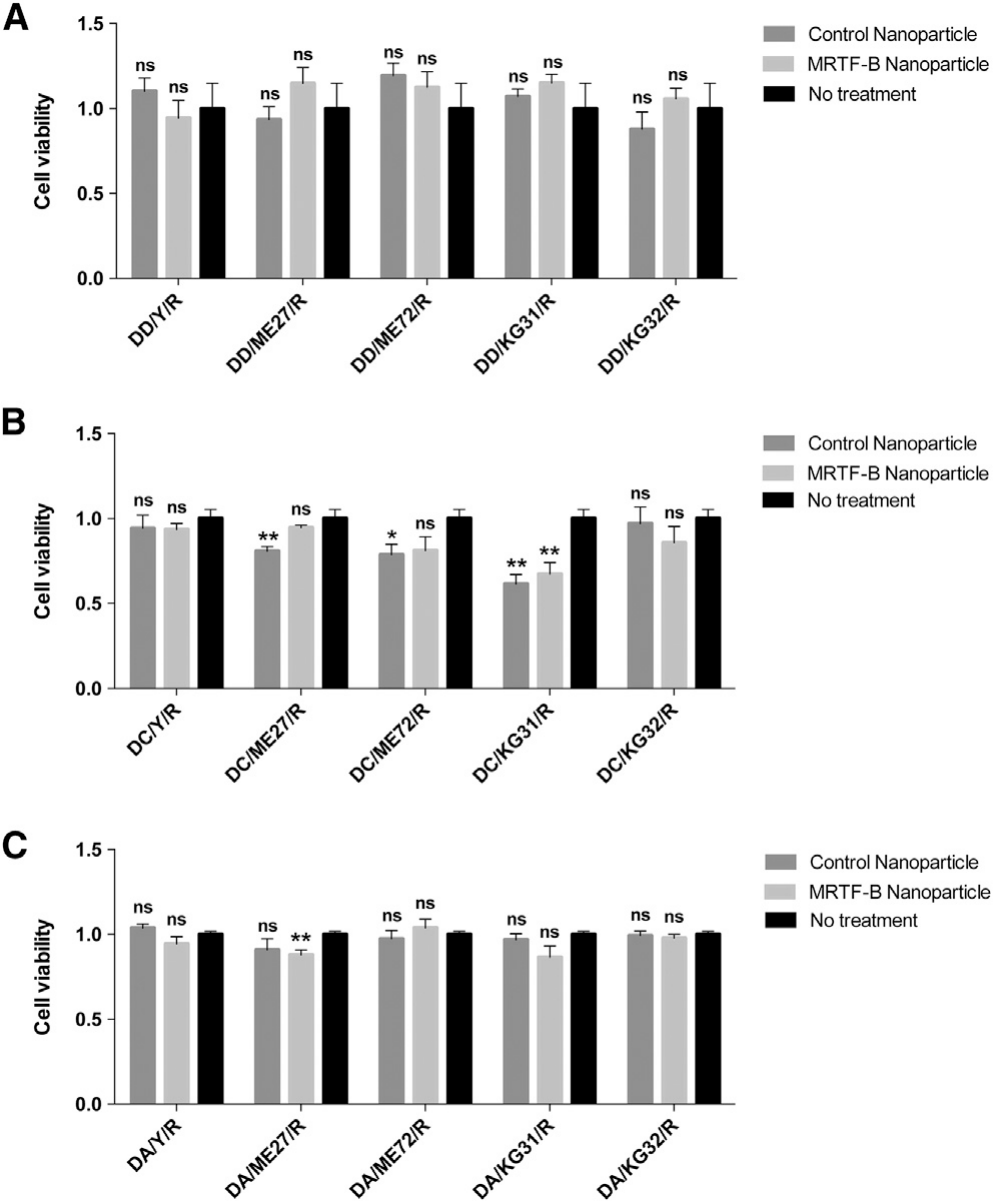
Viability of Human Conjunctival Fibroblasts after Transfection with LPR Nanoparticles for 48 hr (A) LPRs containing DOTMA/DOPE (DD). (B) LPRs containing DOTMA/DOPE + cholesterol (DC). (C) LPRs containing anionic DOPG/DOPE + PEG (DA). Results represent mean ± SEM for six independent replicates. *p < 0.05; **p < 0.01. ns, not significant.

For LPRs containing DD, none of the MRTF-B siRNA-loaded nanoparticles were cytotoxic in human conjunctival fibroblasts compared to control nanoparticles or untreated cells (Figure 3A). There was a statistically significant decrease in cell viability for LPRs containing DC-peptide ME27-control siRNA (p = 0.007), DC-peptide ME72-control siRNA (p = 0.041), DC-peptide KG31-control siRNA (p = 0.001), and DC-peptide KG31-MRTF-B siRNA (p = 0.008) (Figure 3B).

For LPRs containing DA, there was also no statistically significant decrease in cell viability in human conjunctival fibroblasts compared to untreated cells, except for DA-peptide ME27-MRTFB siRNA (p = 0.004) (Figure 3C). Our results show that the cationic LPR formulation of DOTMA/DOPE with the targeting peptide Y (LYR) was the most efficient in *MRTF-B* gene silencing (52.7 ± 2.7%) and the least cytotoxic in human conjunctival fibroblasts; therefore, this formulation was used in all subsequent experiments.

### Biophysical Characterization and Silencing Effect of LYR Nanoparticles in Rabbit Conjunctival Fibroblasts

**Figure 4:**
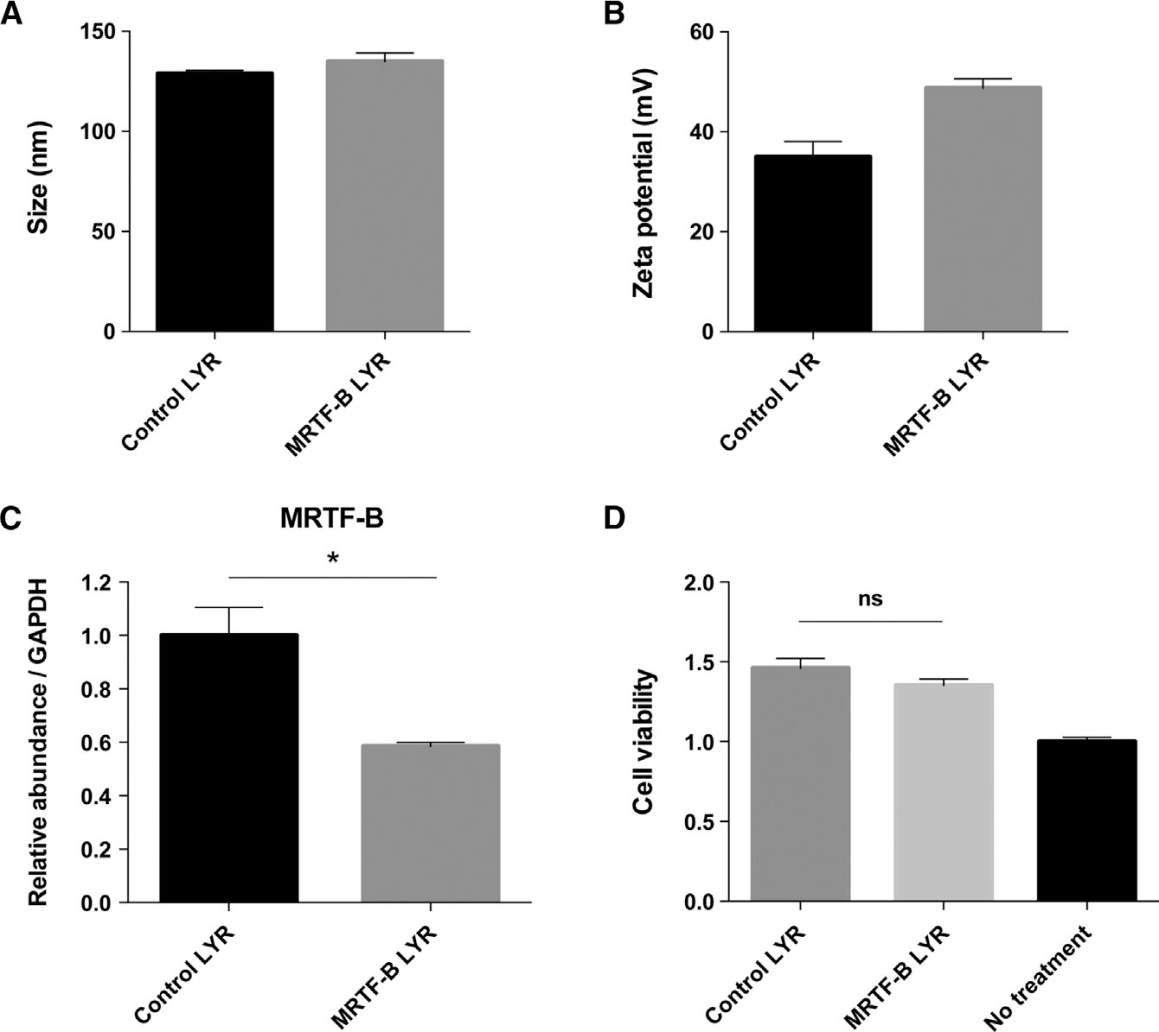
*In Vitro* Transfections of Rabbit Conjunctival Fibroblasts Using LYR and Rabbit siRNA Sequences (A) Size in nanometers. (B) Zeta potential in millivolts. Results represent mean ± SEM. (C) mRNA levels were normalized relative to that of GAPDH, and the results represent mean ± SEM for triplicate experiments. *p < 0.05. (D) Cell viability after transfection for 48 hr. Results represent mean ± SEM for six independent replicates. ns, not significant.

The LYR nanoparticles containing control and rabbit MRTF-B siRNAs had a mean size (±SEM) of 128.8 ± 1.7 nm and 134.9 ± 4.3 nm, respectively (Figure 4A). The LYR nanoparticles containing control siRNA (35.0 ± 3.1 mV) and rabbit MRTF-B siRNA (48.7 ± 1.9 mV) were both strongly cationic (Figure 4B). The PDIs were 0.36 ± 0.01 and 0.41 ± 0.01 for control LYR and rabbit MRTF-B LYR nanoparticles, respectively, indicating that the populations were not monodisperse. MRTF-B LYR nanoparticles decreased *MRTF-B* gene expression in rabbit conjunctival fibroblasts by 41.5% (p = 0.018) compared to control LYR nanoparticles (Figure 4C). MRTF-B LYR nanoparticles also did not have any significant effect on cell viability in rabbit conjunctival fibroblasts compared to control LYR nanoparticles (p = 0.168) (Figure 4D).

### *In Vivo* Administration of LYR Nanoparticles Increases the Long-Term Success of Surgery in a Rabbit Model of Glaucoma Filtration Surgery

**Figure 5:**
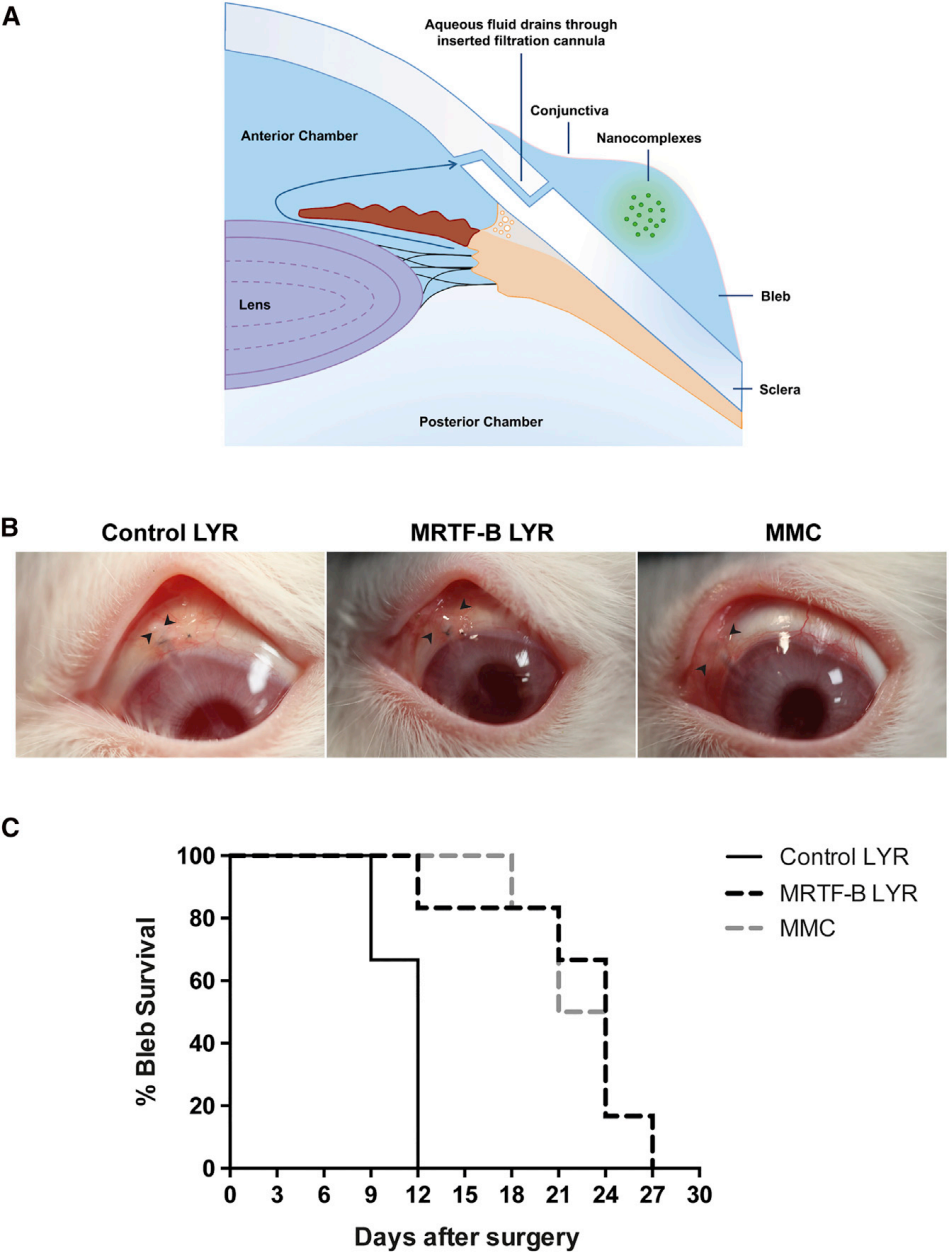
Effect of LYR Nanoparticle Formulations on a Rabbit Model of Glaucoma Filtration Surgery (A) Schematic diagram to illustrate aqueous fluid draining through inserted filtration cannula and subconjunctival administration of nanocomplexes. (B) Morphology of blebs after surgery and treatment with MRTF-B LYR nanoparticles, control nanoparticles, or mitomycin-C (MMC). Arrowheads indicate bleb edges. (C) Kaplan-Meier graph comparing the bleb survival between MRTF-B LYR nanoparticles (n = 6), control LYR nanoparticles (n = 6), and MMC (n = 6).

We used an established and clinically validated rabbit model of experimental GFS to investigate the effects of LYR nanoparticles containing MRTF-B siRNA on wound healing in the conjunctiva.^25^, ^26^ Subconjunctival scarring after GFS is one of the most aggressive models of scar tissue formation, and failure of surgery is due to excessive scarring. A bleb arises when a filtration cannula is inserted during the surgery and drains aqueous fluid from the anterior chamber of the eye to under the conjunctiva (Figure 5A). The primary efficacy endpoint was bleb survival, as this is indicative of the long-term opening of the filtration pathway created during surgery. Bleb failure was defined as the appearance of a flat, scarred, and vascularized bleb associated with a deep anterior chamber. We tested the LYR formulation *in vivo*, as it was non-cytotoxic and the most efficient in terms of *MRTF-B* gene silencing in human conjunctival fibroblasts. Bleb survival was doubled from a mean (±SEM) 11.0 ± 0.6 days for control LYR nanoparticles to 22.0 ± 2.1 days for MRTF-B LYR nanoparticles (p = 0.005), and to 22.5 ± 1.3 days for MMC (p = 0.001) (Figures 5B and 5C).

### LYR Nanoparticles Decrease Conjunctival Scarring and Do Not Cause Any Local or Systemic Side Effects

**Figure 6:**
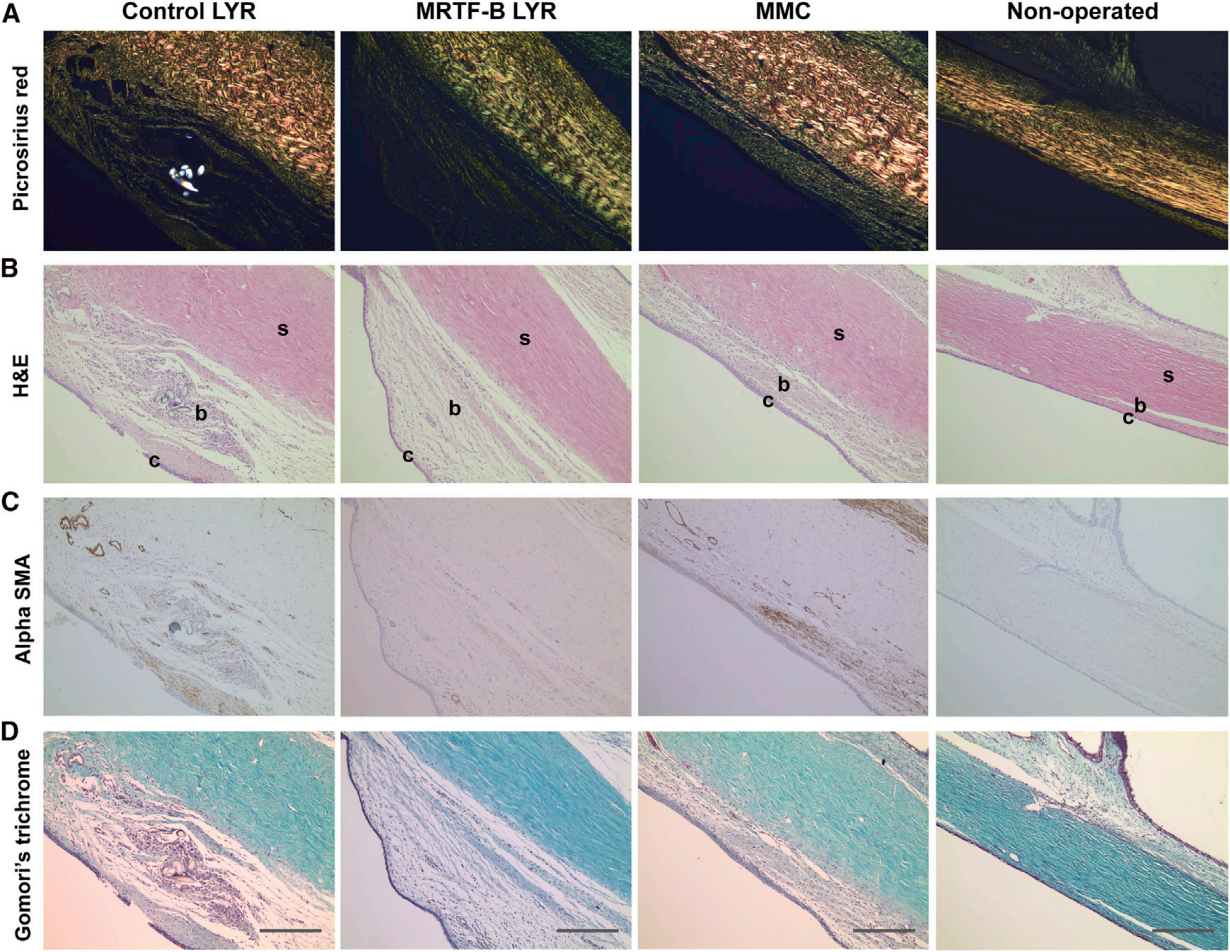
Histology of Rabbit Conjunctival Tissues The left operated eyes were compared to the right non-operated eyes that were used as controls for normal conjunctival tissue. c, conjunctiva; b, subconjunctival space; s, sclera. (A) Picrosirius red; (B) H&E; (C) αSMA; and (D) Gomori’s trichrome stain. Scale bars, 100 μm.

The MRTF-B LYR nanoparticles significantly decreased the scar tissue formation in the rabbit conjunctiva compared to control nanoparticles (Figure 6A). Total cellularity also remained significantly increased in the control LYR nanoparticle group compared to the MRTF-B LYR nanoparticles (Figure 6B). In addition, MRTF-B LYR nanoparticles decreased the expression of alpha-smooth muscle actin (αSMA) by cells, suggesting the presence of fewer myofibroblasts (Figure 6C). Newly laid extracellular matrix and collagen were present to a greater degree in the control LYR nanoparticle group compared to the MRTF-B LYR nanoparticles (Figure 6D).

**Figure 7:**
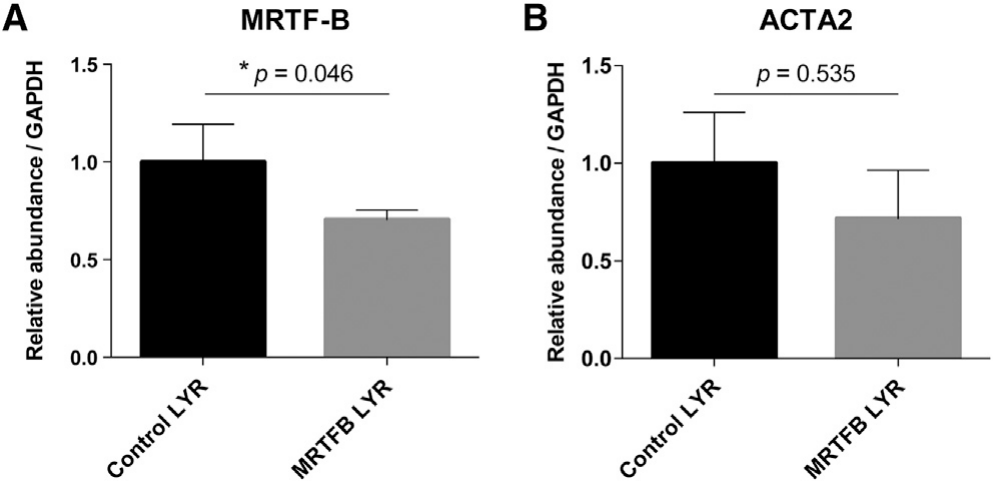
Gene Silencing of *MRTF-B* and *ACTA2* in Rabbit Conjunctival Tissues after Subconjunctival Injection of MRTF-B LYR Nanoparticles (A) *MRTF-B*. (B) *ACTA2*. mRNA levels were normalized relative to that of GAPDH, and the results represent mean ± SEM for triplicate experiments.

We further tested the silencing effect of MRTF-B LYR nanoparticles on *MRTF-B* gene expression *in vivo* in rabbit conjunctival tissues. MRTF-B LYR nanoparticles decreased the *MRTF-B* gene expression by 29.6% in rabbit conjunctival tissues compared to control LYR nanoparticles (p = 0.046) (Figure 7A). MRTF-B LYR nanoparticles also decreased the *ACTA2* gene expression in rabbit conjunctival tissues compared to control LYR nanoparticles; however, this was not statistically significant (p = 0.535) (Figure 7B). In addition, none of the rabbits showed any signs of ocular or systemic toxicity during the study.

## Discussion

This study demonstrates proof of concept that *MRTF-B* targeting by siRNA is an effective application of genetic inhibition to prolong bleb survival and to prevent conjunctival fibrosis after experimental GFS. Moreover, we have shown that the MRTF-B siRNA-loaded nanoparticles are safe to use *in vivo* in the conjunctiva and do not lead to any local or systemic adverse side effects. Our previous studies have also shown that direct injection is an efficient route of administration of nanoparticles; for example, direct injection into subcutaneous tumors^27^, ^28^ and convection enhanced delivery through a cannula directly into the brain.^21^, ^29^

Potential advantages of nanoparticle-mediated siRNA therapy over drugs, such as MMC, include its cell specificity, potency, and duration.^30^ There has been increasing interest in developing siRNA-based therapeutics in herpetic stromal keratitis,^31^ retinoblastoma,^32^ ocular inflammation,^33^ non-arteritic anterior ischemic optic neuropathy,^34^ age-related macular degeneration,^35^, ^36^ and to lower the intraocular pressure in glaucoma.^37^ Other research groups are also developing different types of nanoparticles to modulate wound healing in the eye.^38^, ^39^ Layer-by-layer nanoparticles (LbL-NPs) represent an efficient delivery vehicle for the siRNA silencing of secreted protein, acidic, and rich in cysteine (SPARC).^38^ Cationic nano-copolymers combined with IKKβ siRNA—CS-g-(PEI-b-mPEG)/IKKβ-siRNA—also increased bleb survival and decreased subconjunctival scarring in a monkey model of GFS.^39^ There were no toxic side effects reported with the layer-by-layer nanoparticles and cationic nano-copolymers.

By designing a series of peptides, the goal was to understand how these different interactions affect gene delivery outcomes. Peptide Y was identified by biopanning a phage peptide library and closely resembles part of a targeting protein expressed by the intracellular pathogen *Legionella pneumophila*.^40^, ^41^ Although the identity of the receptor is still unknown, we have shown that peptide Y mediates the targeted delivery of siRNA in nanocomplexes to different tissues, including cells of neuronal origin,^41^, ^42^ lung cells,^22^, ^28^, ^43^ primary vascular cells, and rabbit aorta.^40^, ^44^ ME27 is a cleavable peptide that contains a tripeptide Arg-Gly-Asp (RGD) motif that targets integrins—particularly α_v_β_3_, α_v_β_5,_ and α_5_β_1_—and these surface integrin receptors are abundantly expressed on human eye fibroblasts.^45^

The targeting peptide KG31 consists of a hydrophobic Y, and KG32 contains a cleavable Y. We have previously synthesized peptides containing hydrophobic amino acids as spacers between a K_16_ moiety and an integrin-targeting motif. Our previous results had also shown that vectors containing peptides incorporating linkers that are partly hydrophobic demonstrated improved transfection properties, which is likely due to the improved accessibility of the integrin-binding motif.^46^ We also hypothesized that, by adding the cleavable linker RVRR in KG32, as is the case in peptide ME27, this would promote processing of peptide components of receptor-targeted nanocomplex formulations. RVRR peptide motifs are cleavable by the endosomal enzymes furin and cathepsin B.^47^ We have previously shown *in vitro* that peptides with cleavable linkers displayed improved transfection efficiencies compared to their non-cleavable peptide homologs.^47^ The reason might be because endosomal cleavage of the peptides can lead to disengagement of the nanoparticle from the receptor, allowing more efficient release of the particle from the endosomal membrane, or removal of a peptide layer may make the particle smaller and, thus, more readily transported through the cytoplasm and the nuclear envelope.

In this study, cationic formulations using targeting peptides Y, ME27, KG31, and KG32 produced higher transfection efficiencies in human conjunctival fibroblasts than their non-targeting counterparts containing peptide ME72, suggesting receptor-specific transfection. Our previous study has also shown that receptor-targeted nanoparticles using peptides Y and ME27 achieved higher gene silencing efficiencies in transfecting conjunctival fibroblasts than nanoparticles with the non-targeting peptide K_16_ or with no peptides.^18^ Even though the new targeting peptides KG31 and KG32 increased the transfection efficiency and *MRTF-B* gene silencing in human conjunctival fibroblasts compared to the non-targeting peptide, thereby supporting our aforementioned hypothesis, they did not, however, provide improved gene silencing over peptide Y.

Several studies have also reported a size-dependent cytotoxicity of nanoparticles.^48^, ^49^, ^50^ In our study, LPRs containing DD were around 120 nm, with a relatively low polydispersity index (PDI) (0.37), and had no significant effect on cell viability in human conjunctival fibroblasts. On the other hand, LPRs containing DC had significantly larger particle size (around 235 nm) and a high PDI (0.84), indicating a broad particle size distribution, and were cytotoxic to human conjunctival fibroblasts. LPRs containing DA were around 102 nm, with a low PDI (0.25), and had no significant effect on cell viability.^21^, ^29^

Previous studies have shown increased targeted transfection efficiency, superior distribution, and decreased inflammatory response of anionic PEGylated nanocomplexes *in vivo* compared to their homologous cationic formulations.^21^, ^22^, ^29^ However, our results show that anionic PEGylated formulations did not have any significant effect *in vitro* on *MRTF-B* gene silencing in human conjunctival fibroblasts. One reason might be that the previous studies were conducted in cell lines as opposed to the primary human conjunctival fibroblasts used here. Therefore, on the basis of the *in vitro* silencing results, we did not progress to *in vivo* studies with the anionic formulations that could have shown possibly better silencing than those in the tissue culture studies and could have been more comparable to that of the cationic counterparts. Future studies could involve the use of reduced PEGylation (e.g., 0.5% or 1%) to determine whether that will enhance the silencing efficiency of anionic nanoparticles in human conjunctival fibroblasts.

Due to the positive zeta potential of *MRTF-B* LYR nanoparticles, interaction with proteins and other components *in vivo* can lead to aggregation. We did not observe any aggregation *in vivo*, and we achieved a 29.6% *MRTF-B* gene silencing in rabbit conjunctival tissues after a single subconjunctival injection of MRTF-B LYR nanoparticles compared to control nanoparticles. This level of silencing was associated with an increase in bleb survival and a decrease in conjunctival scarring in a rabbit model of experimental GFS. MMC did not have any significant advantage over LYR nanoparticles. MMC works as an anti-scarring drug by causing widespread apoptosis of fibroblasts and is not known to have an effect on the *MRTF-B* gene.^3^ LYR design and dosage can also be further optimized and improved in future studies. We have previously shown that a 70% *MRTF-B* gene silencing in human conjunctival fibroblasts after a single transfection treatment completely blocked matrix contraction in a 7-day collagen contraction assay.^18^

One of the major challenges in developing novel anti-fibrotic drugs in GFS is to design long-acting drugs that would only need to be applied once at the time of surgery.^51^ Future work will focus on optimizing the best concentration and timing of injections to achieve long-acting and sustained gene silencing to prevent conjunctival fibrosis. For future clinical development, it will also be important to develop protocols for large-scale mixing of the lipid, peptide, and siRNA components using Good Manufacturing Practice (GMP) materials and processes.^52^, ^53^ Such methods include microfluidic mixing and more standard, in-line mixing methodologies as reported for lipid and nucleic acid formulations.^54^

### Conclusions

In this study, we have shown that receptor-targeted liposome-peptide-siRNA nanoparticles represent a safe and efficient siRNA delivery system that could be used to prolong bleb survival and to prevent conjunctival fibrosis after glaucoma filtration surgery by targeting the *MRTF-B* gene as well as other potential gene targets associated with fibrosis.

## Materials and Methods

### Materials

DOTMA, DOPE, DOPG, DPPE PEG 2000, and cholesterol were purchased from Avanti Polar Lipids (Alabaster, AL, USA). The structures of the lipids, peptides, and siRNAs are shown in Table 1. Peptides Y (K_16_GACYGLPHKFCG), ME27 (K_16_RVRRGACRGDCLG), ME72 (K_16_RVRRGACRGECLG), KG31 (K_16_RXSXGACYGLPHKFCG, Hydrophobic Y, X = epsilon-aminohexanoic acid), and KG32 (K_16_RVRRGACYGLPHKFCG, Cleavable Y) were synthesized by ChinaPeptides (Shanghai, China) and AMS Biotechnology (Abingdon, UK). The siRNAs used in this study were custom synthesized and purchased from Dharmacon (Cambridge, UK). The siRNAs were dissolved in sterile RNase-free water at a concentration of 2.5 mg/mL.

**Table 1:** Structures of the Different Lipids and Sequences of Peptides and siRNAs

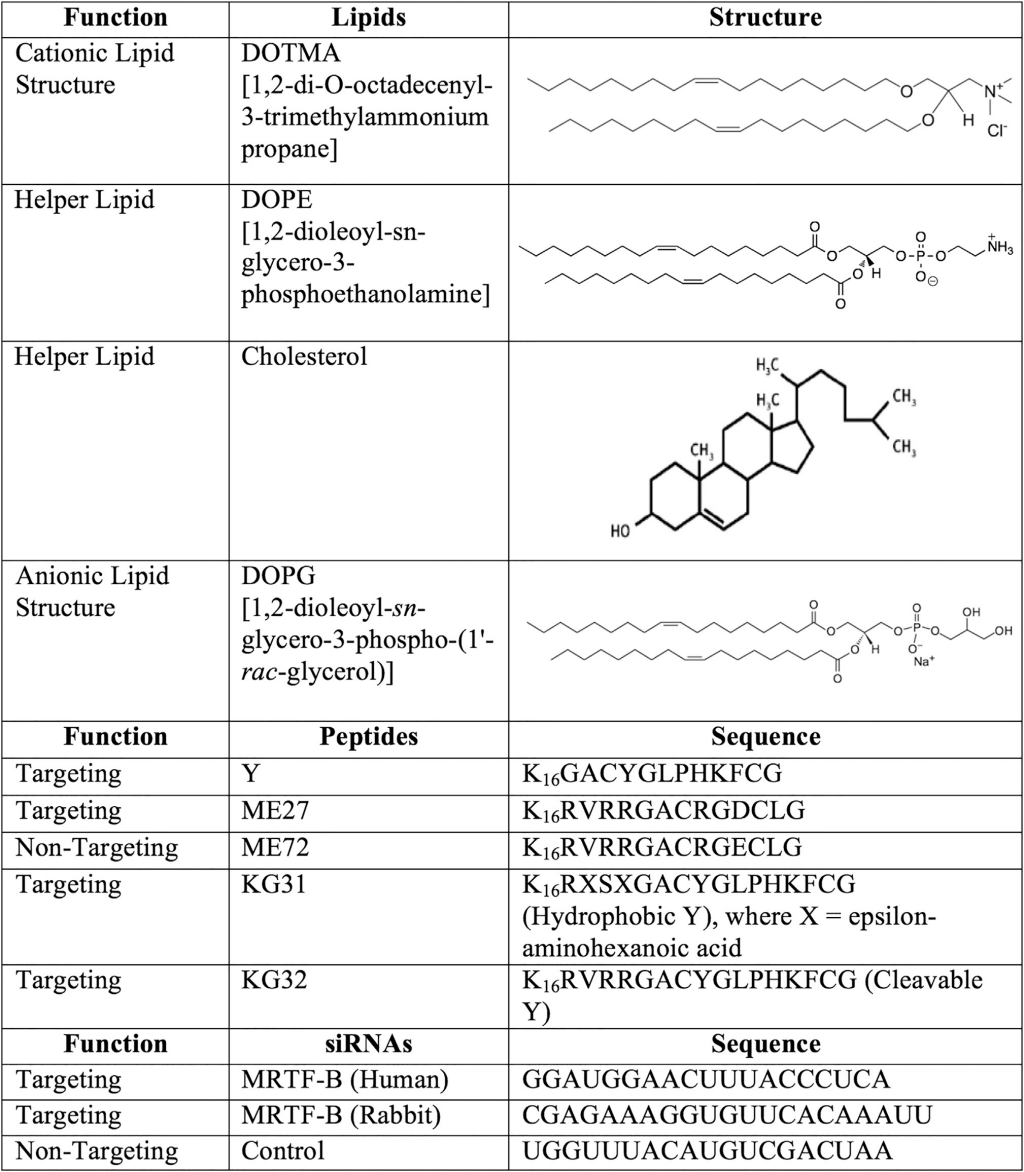

### Liposome Preparation

Cationic liposomes made were as follows: DD at a 1:1 molar ratio and DC at a molar ratio of 47.5:47.5:5 mol %. Anionic liposomes made were DA at a molar ratio of 49.5:49.5:1 mol %. The lipids were dissolved in chloroform at 10 mg/mL, and a lipid film was produced in a rotary evaporator by slowly evaporating the chloroform. Lipids were rehydrated with sterile RNase-free water while being constantly rotated overnight and then sonicated in a water bath to reduce their size.

### Cell Culture

Human conjunctival fibroblasts were grown from donor human conjunctiva, and informed consent was obtained from all subjects. Rabbit conjunctival fibroblasts were grown from New Zealand white rabbit conjunctival tissues. The fibroblasts were maintained in DMEM (Invitrogen) with 10% fetal bovine serum (FBS), 100 U/mL penicillin, 100 mg/mL streptomycin, and 2 nM L-glutamine, in tissue culture incubators with 5% CO_2_ and 95% humidity. Fibroblasts between passages 2 and 8 were used in the experiments. All experimental protocols were approved by the London-Dulwich Research Ethics Committee (REC 10/H0808/127) and the institutional approval committee at the University College London Institute of Ophthalmology.

### Nanoparticle Formulation

LPRs containing DD and LPRs containing DC were prepared at a weight ratio of 1:4:1 (liposome:peptide:siRNA) by first mixing the liposome with the peptide, followed by the addition of siRNA. The mixture was incubated at room temperature (25°C) for 1 hr to allow complex formation. OptiMEM (Life Technologies, Paisley, Scotland, UK) was then added to give a final siRNA concentration of 50 nM. LPRs containing DA were prepared at a weight ratio of 19:2.7:1 (liposome:peptide:siRNA) by first mixing the peptide with the siRNA and then incubating for 30 min at room temperature. The liposome was then mixed with the peptide-siRNA and incubated for a further 30 min.

### Nanoparticle Size and Zeta Potential

Nanoparticle size and zeta potential were determined by dynamic light scattering (DLS) and laser Doppler anemometry, respectively, using a Nano ZS Zetasizer (Malvern Instruments, Malvern, UK) with the following specifications: automatic sampling time of 10 measurements per sample; refractive index of 1.330; dielectric constant, 78.5; viscosity, 0.8872 cP; and temperature of 25°C. Zeta potential settings were calibrated against the standard (−68 ± 6.8 mV). Triplicate measurements were performed for each sample, and the results were analyzed using the software provided by the manufacturer (DTS, v5.03).

### TEM

The nanoparticles were applied onto a 300-mesh copper grid coated with a Formvar/carbon support film (Agar Scientific, Stansted, UK) and processed as previously described.^21^ The samples were negatively stained with 1% uranyl acetate for 2–3 s before blotting with filter paper and air drying. Imaging was performed with a Philips CM120 BioTwin transmission electron microscope and operated at an accelerating voltage of 120 kV. Images were captured using an AMT 5MP digital TEM camera (Deben UK, Suffolk, UK).

### *In Vitro* Transfection

The human and rabbit conjunctival fibroblasts were seeded at 1 × 10^5^ cells per well in 6-well plates (Falcon; Fisher Scientific UK) and incubated for 24 hr before the nanocomplexes were added. The cells were incubated with the nanocomplexes in OptiMEM for 4 hr at 37°C. The medium containing the nanocomplexes was then replaced by fresh growth medium, and the cells were incubated for a further 48 hr at 37°C.

### Real-Time qPCR

The human and rabbit conjunctival fibroblasts were lysed for RNA extraction using the RNeasy Mini Kit (QIAGEN, Manchester, UK) according to the manufacturer’s instructions. The rabbit conjunctival tissues were mechanically dispersed, and RNA was also extracted using the RNeasy Mini Kit (QIAGEN, Manchester, UK). Real-time qPCR reactions were performed using a SensiFAST SYBR Hi-ROX One-Step Mix (Bioline, London, UK) on a CFX Real-Time PCR Detection System (Bio-Rad, Hemel Hempstead, UK). The real-time qPCR assay conditions were as follows: stage 1, 45°C for 20 min; stage 2, 95°C for 3 min; stage 3, 95°C for 10 s and then 60°C for 25 s; repeated 40 times. The human TaqMan gene expression assays were MRTF-B/MKL2 (Hs00401867_m1) and GAPDH (Hs02758991_g1) (Thermo Fisher Scientific, Basingstoke, UK). The rabbit primers were as follows: MRTF-B, 5′-CTCCGATGTGGGTTTATGGGT-3′, 3′-GGAAGTGGCATCAGGACAGT-5′; ACTA2, 5′-TCCACCGCAAATGCTTCTAAGT-3′, 3′-ATGAGTCAGAGCTTTGGATAGGC-5′; GAPDH, 5′-CGAGACACGATGGTGAAGGT-3′, 3′-CCAGCATCACCCCACTTGAT-5′. All mRNA values were normalized relative to that of GAPDH, and triplicate experiments were performed for each condition.

### Cell Proliferation Assay

The human and rabbit conjunctival fibroblasts were seeded at 0.625 × 10^4^ cells per well in 96-well plates (Falcon; Fisher Scientific UK). The cells were transfected with six replicates for each nanoparticle formulation, and cell viability was measured using the CellTiter 96 AQueous One Solution Cell Proliferation Assay (Promega, Southampton, UK). The medium was replaced with 100 μL fresh growth medium per well, and 20 μL of the CellTiter 96 AQueous One Solution was added to each well. The cells were incubated for 2 hr, and absorbance at 540 nm was measured using a FLUOstar Optima (BMG LABTECH). Cell viability for each formulation was expressed as a percentage of the viability of control cells.

### Rabbit GFS Model of Scar Tissue Formation

All animal procedures were performed in accordance with the Association of Research in Vision and Ophthalmology (ARVO) Statement for the Use of Animals in Ophthalmic and Vision Research, and all animal experimental protocols were approved by the Home Office UK (PPL 70/8074). Eighteen female New Zealand white rabbits (1.5–2 kg, 10–12 weeks old, Envigo, Cambridgeshire, UK) underwent experimental GFS to the left eye under general anesthesia.^25^, ^26^ A superonasal fornix-based conjunctival flap was raised behind the limbus, and a micro-vitreoretinal blade (20G, 0.90 mm, Surgistar USA) was used to make a partial-thickness scleral tunnel to the corneal stroma. A 22G, 25-mm intravenous cannula was passed through the tunnel, and the needle was removed once it was visible in the cornea. The cannula was advanced into the mid-pupillary area, trimmed at its scleral edge, and fixed to the scleral surface using a 10-0 nylon suture (Alcon, Fort Worth, TX, USA). The conjunctival incision was closed using two interrupted 10-0 nylon sutures. In a randomized, prospective, single-masked observer study, the rabbits received an intraoperative injection of 0.2 mg/mL MMC (n = 6) or a postoperative subconjunctival injection of 25 μg in 100 μL MRTF-B LYR nanoparticles (n = 6) or control LYR nanoparticles (n = 6).

### Post-operative Clinical Examination

The animals were examined every 3 days by a single-masked blinded researcher. Bleb width and length were measured using calipers, and intraocular pressures were measured using a tonovet (Icare, Vantaa, Finland). The primary efficacy endpoint was bleb survival, as this is indicative of the long-term patency of the filtration pathway created during surgery. Bleb failure was defined as the appearance of a flat, scarred, and vascularized bleb associated with a deep anterior chamber. All animals were also evaluated for signs of local or systemic toxicity during the study.

### Histologic Analysis

The animals were sacrificed on day 30, and both eyes were enucleated. The eyes were fixed in formalin and embedded in paraffin, and sequential 4-μm tissue sections were cut. The sections were stained with H&E (for cellularity and inflammatory cells), Gomori’s trichrome (for collagen), picrosirius red (for degree of fibrosis), and αSMA using a primary monoclonal mouse anti-human αSMA antibody (Clone 1A4; Dako, High Wycombe, UK) and a biotinylated secondary antibody (rabbit anti-mouse; Dako). All the left operated eyes were compared to the right non-operated eyes that were used as controls for normal conjunctival tissue.

### Statistical Analysis

All graphs display the mean and SEM. Statistical analysis was performed using the Student’s t test to calculate statistically significant differences and p values. Survival analysis for bleb was performed using the Kaplan-Meier log-rank test. Statistically significant differences were expressed as *p < 0.05, **p < 0.01, and ***p < 0.001.

## Author Contributions

C.Y.-W.-M. designed the experiments, obtained funding for research, conducted the research, supervised staff, and wrote the manuscript. O.F., A.D.T., and S.A. performed the experiments, analyzed the data, and contributed to the manuscript. S.L.H., P.T.K., and S.B. contributed to the research and to the manuscript.

## Conflicts of Interest

The authors have no conflicts of interest.
